# Abnormal lacuno-canalicular network and negative correlation between serum osteocalcin and Cobb angle indicate abnormal osteocyte function in adolescent idiopathic scoliosis

**DOI:** 10.1096/fj.201901227R

**Published:** 2019-10-18

**Authors:** Huanxiong Chen, Jiajun Zhang, Yujia Wang, Ka-Yee Cheuk, Alec L. H. Hung, Tsz-Ping Lam, Yong Qiu, Jian Q. Feng, Wayne Y. W. Lee, Jack C. Y. Cheng

**Affiliations:** * Department of Spine and Osteopathic Surgery, The First Affiliated Hospital of Hainan Medical University, Hai-kou, China;; † Department of Orthopaedics and Traumatology, S. H. Ho Scoliosis Research Laboratory, The Chinese University of Hong Kong, Shatin, NT, Hong Kong, China;; ‡ Joint Scoliosis Research Center of The Chinese University of Hong Kong–Nanjing University, The Chinese University of Hong Kong, Hong Kong, China;; § Spine Surgery, Nanjing Drum Tower Hospital, Nanjing University, Nanjing, China;; ¶ Department of Biomedical Sciences, Texas A&M College of Dentistry, Dallas, Texas, USA

**Keywords:** bone biopsy, bone serum markers, scanning electron microscopy, calcium to phosphorous ratio, microindentation

## Abstract

Adolescent idiopathic scoliosis (AIS) is a prevalent spinal deformity occurring during peripubertal growth period that affects 1–4% of adolescents globally without clear etiopathogenetic mechanism. Low bone mineral density is an independent and significant prognostic factor for curve progression. Currently, the cause underlying low bone mass in AIS remains elusive. Osteocytes play an important role in bone metabolism and mineral homeostasis, but its role in AIS has not been studied. In the present study, iliac bone tissues were harvested from 21 patients with AIS (mean age of 14.3 ± 2.20 yr old) with a mean Cobb angle of 55.6 ± 10.61° and 13 non-AIS controls (mean age of 16.5 ± 4.79 yr old) intraoperatively. Acid-etched scanning electron microscopy (SEM) images of AIS demonstrated abnormal osteocytes that were more rounded and cobblestone-like in shape and were aligned in irregular clusters with shorter and disorganized canaliculi. Further quantitative analysis with FITC-Imaris technique showed a significant reduction in the canalicular number and length as well as an increase in lacunar volume and area in AIS. SEM with energy-dispersive X-ray spectroscopy analysis demonstrated a lower calcium-to-phosphorus ratio at the perilacunar/canalicular region. Moreover, microindentaion results revealed lower values of Vickers hardness and elastic modulus in AIS when compared with controls. In addition, in the parallel study of 99 AIS (27 with severe Cobb angle of 65.8 ± 14.1° and 72 with mild Cobb angle of 26.6 ± 9.1°) with different curve severity, the serum osteocalcin level was found to be significantly and negatively associated with the Cobb angle. In summary, the findings in this series of studies demonstrated the potential link of abnormal osteocyte lacuno-canalicular network structure and function to the observed abnormal bone mineralization in AIS, which may shed light on etiopathogenesis of AIS.—Chen, H., Zhang, J., Wang, Y., Cheuk, K.-Y., Hung, A. L. H., Lam, T.-P., Qiu, Y., Feng, J. Q., Lee, W. Y. W., Cheng, J. C. Y. Abnormal lacuno-canalicular network and negative correlation between serum osteocalcin and Cobb angle indicate abnormal osteocyte function in adolescent idiopathic scoliosis.

Scoliosis is defined as structural deformity of the spine and is diagnosed by measuring the Cobb angle (a line drawn parallel to the superior endplate of the upper vertebra and a line drawn parallel to the inferior endplate of the low vertebra of the same curve). Adolescent idiopathic scoliosis (AIS) is the most common type of scoliosis that affects adolescents between the ages of 10 and 13 with a prevalence rate of 1–4% worldwide. In general, about 10% of patients with AIS with progressive deformity require treatment including bracing for a Cobb angle ≥20° and surgical spinal fusion for a Cobb angle ≥45° (1, 2). Currently available treatments, including bracing and instrumental surgical correction, are targeting the anatomic spinal abnormalities instead of the cause owing to unclear pathogenesis. Genetic factor has been hypothesized to play an important role in the pathogenesis as indicated by a higher occurrence rate in monozygotic twins (73%) than in dizygotic twins (36%) (3), but considerable discordance in the pattern, level, and severity of curve deformity in monozygotic twins’ studies suggests the presence of other pathogenic factors (4, 5).

AIS subjects are associated with low bone mass. Their siblings with normal spines had normal bone mass (6). By dual-energy X-ray absorptiometry (DXA), the negative relationship between bone mass and spinal deformity has been reported in different ethnic groups (7–10). Recent high-resolution peripheral quantitative computed tomography studies at distal radius confirmed lower cortical and trabecular bone volume and poorer bone mechanical properties in AIS (11–13). Areal bone mineral density (aBMD) at femoral neck and cortical volumetric BMD at distal radius were later found to be prognostic factors for curve progression (14, 15). Lower bone mass is speculated to increase the tendency of vertebral bone wedging, which contributes to curve progression in AIS (16). The interplay between abnormal bone quality and curve progression in AIS could be a novel therapeutic target.

Osteocytes, descendant of osteoblasts, are the most abundant bone cells (>90%) that regulate bone metabolism. Osteocytes interconnect with neighboring osteocytes *via* dendritic processes protruding from their cell bodies. Because osteocytes are embedded in a mineralized matrix, the interconnecting network is characterized as lacuno-canalicular network (LCN), which is important for mechanosensation and mechanotransduction (17). Previous bone biopsy histomorphometry studies revealed lower bone mineralization, a greater osteoblast number, and a lower number of osteocytes in patients with AIS and with severe spinal deformity (18, 19), suggesting the likelihood of impaired osteoblasts to osteocytes differentiation. Recently, our cellular study first reported defective osteocyte activities and lower expression of dendritic markers in AIS owing to overexpression of miRNA-145 (20). LCN is essential for maintaining osteocytes viability and functions (21). Studies in other pathologic conditions showed that alteration in osteocyte LCN is associated with changes in the bone matrix’s composition and mechanical competence (22, 23). At the macroscopic level, reduction in osteocyte activity and LCN structure have implications for lower bone mass, deranged bone microarchitecture, and higher fracture rate (24–26), but there is yet no direct evidence of structural abnormality of osteocyte LCN in AIS at bone tissue level.

This study aimed to investigate whether there is any structural defect of osteocyte LCN in AIS bone tissues collected from a surgical case. We also determined the calcium-to-phosphorous level and Vickers hardness and elastic modulus to provide a better understanding of the bone quality in AIS and control group. Given that bone tissue from patients with AIS and with mild to moderate severity is very scarce (this group of patients do not request surgical correction), an alternative approach using serum samples from a cross-sectional cohort with mixed curvature was used to explore if osteocyte activity is associated with curve severity.

## MATERIALS AND METHODS

### Subjects

Clinical ethical approval in compliance with the Declaration of Helsinki was obtained from our institutional review board. Informed consent was obtained from all subjects or their legal guardians. The *ex vivo* study consisted of 20 Chinese girls with AIS; all had severe progressive curves with a Cobb angle over 45° and required surgical instrumentation and posterior spinal fusion. Basic anthropometric data, the type of curve, time of occurrence, and progression pattern were recorded. Exclusion criteria include: *1*) congenital scoliosis, *2*) neuromuscular scoliosis, *3*) scoliosis of metabolic etiology, *4*) scoliosis with skeletal dysplasia, or *5*) scoliosis with known endocrine and connective tissue abnormalities. Thirteen non-AIS Chinese adolescents who required orthopedic bone related reconstructive surgery were recruited. The control subjects were carefully assessed by 2 senior orthopedic clinicians to rule out scoliosis and other known bone metabolic diseases.

In the serological study, 99 AIS girls with different curve types, curve severities (Cobb angle ranged from 14° to 86°), and corresponding treatments (19 cases with observation, 47 cases with bracing, and 33 cases with surgery) were randomly recruited at the same scoliosis clinic. The diagnosis of AIS was confirmed clinically by at least 2 senior orthopedic surgeons and radiologically with standing full-spine posteroanterior X-ray. Subjects with the abovementioned exclusion criteria were not recruited. A total of 31 healthy girls of a similar age were recruited randomly from local secondary schools to serve as controls. They were examined clinically by the experienced orthopedic surgeons to exclude for spinal deformities. All the subjects with congenital deformities, neuromuscular diseases, autoimmune disorders, endocrine disturbances, or medical conditions that affect bone metabolism were excluded.

### Demographic, anthropometric, and radiologic assessments

Body weight and arm span were measured with standardized stadiometric techniques. Arm span was used for calculating the body mass index [BMI; BMI = body weight (kg)/arm span (m^2^)] to minimize the inaccuracy that is caused by spinal deformity in AIS (27). Tanner stage was used for the assessment of the sexual maturity. For AIS girls, the degree of curvature was measured by the Cobb method in the standard standing posteroanterior radiographs of the whole spine. The Cobb angle of the major curve was measured within a month before or after blood taking. As the axial vertebral rotation of the deformed spine could affect aBMD measurement, aBMD (g/cm^2^) of the femoral neck in AIS was measured instead by DXA (XR-36; Norland Medical Systems, Fort Atkinson, WI, USA) as previously described in Cheng *et al*. (28). A normative aBMD dataset of local Chinese girls was used for the calculation of the age and gender-adjusted *z* score.

### Bone biopsy samples

Trabecular bone tissues were collected from ilium (2 cm anterior to the posterior superior iliac spine) of patients intraoperatively as part of the procedure for taking autograft. In brief, the ilium was surgically exposed and multiple trabecular bone slabs and chips were taken with chisel and osteotomes from the marrow space with preservation of the iliac crest apophysis and inner wall of the ilium. These bone tissues were used as autografts to enhance bony fusion of the respective reconstructive surgery. The surgical procedure was followed by meticulous hemostasis and layered wound closure. For the control, bone biopsies were taken from bone chips or trabecular bone slabs taken intraoperatively as part of the respective surgical procedure. The bone tissues were immediately processed for energy-dispersive X-ray spectroscopy (EDX) and microindentation, and acid-etched scanning electron microscopy (SEM) and FITC-Imaris imaging analysis.

### Methylmethacrylate embedding and sectioning

The bone tissues were fixed in 70% ethanol overnight and then embedded in Technovit 9100 methylmethacrylate (Kulzer, Hanau, Germany) according to the manufacturer’s instructions. The embedded bone tissues were trimmed with a diamond band cutting device (Exakt 300 CP; Exakt Apparatebau, Norderstedt, Germany), then cut into ∼200-μm-thick sections with a diamond inner-hole saw (Leica SP1600; Leica, Wetzlar, Germany). Finally, the 200-μm-thick sections were sanded down to about 150-μm-thick slices with sequential 1200, 2400, and 4000 grit silicon carbide waterproof abrasive paper on a polishing machine (Jean Wirtz Phoenix 4000; Jean Wirtz, Dusseldorf, Germany).

### Backscatter SEM for visualization of lacunae

Bone sections that were 150 μm thick were subjected to gold and palladium coating and examined with a field emission environment SEM (WL30; Thermo Fisher Scientific, Waltham, MA, USA) equipped with a backscatter detector (Thermo Fisher Scientific). Backscatter SEM (BSEM) of the bone sections was taken at various magnifications from ×150 to 2000. All the images were taken in a single-blinded manner.

### Acid-etched resin-cased SEM imaging

Bone sections that were 150 μm thick were immersed in 37% phosphoric acid for 5 s and then in 5% sodium hypochorite for 5 min to remove mineralized and organic matrix to expose the LCN. The etched bone sections were coated with gold and palladium and then examined with SEM (SU8100; Hitachi, Tokyo, Japan) under 5 keV accelerating voltage, 10 μA probe current, a 10-mm working distance, and an image resolution of 1560 × 1920. SEM images of LCN were taken at different magnifications from ×200 to 2500 for the observation of osteocyte distribution, LCN morphology, and degree of connectivity. All the images were taken in a single-blinded manner.

### FITC staining

In the present study, in addition to the acid-etched SEM technique, which provided qualitative data, the FITC-Imaris technique allowed 3-dimensional (3D) quantitative measurement of the LCN. Bone biopsies were fixed in 70% ethanol for 48 h, and dehydrated in ethanol (from 80 to 100%, 2 d each). The samples were then stained in 1% (w/v) FITC isomer I (F7250; MilliporeSigma, Burlington, MA, USA) solution in absolute ethanol for 24 h at room temperature with gentle mixing before embedding in Technovit 9100 and sectioned into 150-μm-thick bone sections as previously described. The bone sections were further sanded down with silicon carbide abrasive paper and polished on a rotating wheel with micropolish suspension (1, 0.25, and 0.05 μm; Buehler, Lake Bluff, IL, USA) to achieve ∼70–100-μm-thick bone sections for confocal microscope imaging. FITC-stained samples were kept from light during preparation and storage.

### Confocal imaging and quantitative analysis of LCN properties

The polished FITC-stained bone sections were mounted on glass slides (Superfrost Plus; Thermo Fisher Scientific) with distilled water as the mounting medium and were covered with 18 × 18-mm coverslips (0.25-mm thickness; VWR, Radnor, PA, USA). Confocal stacking images consisting of 5 or more osteocytes per field of view were obtained in a single-blinded manner with SP8 confocal microscope (Leica) with the following settings: ×63 water immersion lens, 0.5 AU pinhole, 100 Hz, and 1 frame per scan. FITC was excited by a 488-nm argon laser, and emission at 519 ± 5 nm was acquired by a hybrid detector. Stacking images with a thickness of ∼40 μm was obtained with a pixel size of 0.180 × 0.180 × 0.208 µm at a resolution of 1024 × 1024 with linear *z* compensation. The confocal stacking images were constructed into 3D images with Imaris software (v.8.0; Bitplane, Zürich, Switzerland) at the same dimension with a pixel size of 0.180 × 0.180 × 0.208 µm. Only osteocytes with complete cell bodies and canaliculi were selected for analysis. Canaliculi were mapped with the same threshold (0.5–8 µm for filament tracing). The canalicular number, canalicular length, lacunar surface area, lacunar volume, and roundness of lacunae (lengths of major, intermediate, and minor axes) were analyzed as previously described in Ren *et al*. (29).

### Measurement of calcium and phosphorous with SEM-EDX

The local amount of the elements calcium and phosphorus in the bone material was semiquantified with the aforementioned SEM equipped with an IXRF system (IXRF Systems, Austin, TX, USA). First, SEM images of individual lacuna and surrounding region were captured with WL30 (Thermo Fisher Scientific) equipped with a backscatter detector (Thermo Fisher Scientific) at various magnifications from ×150 to 2000. On average, 20 lacunae per bone section were analyzed. In brief, the SEM was operated at a 10-keV accelerating voltage, 10-μA probe current, and 15-mm working distance with a data acquisition time of 130 s as previously described in Wang *et al*. (19). The take-off angle of the SEM-EDX detector was set at 35°. The relative weight of calcium and phosphorus was acquired with built-in software, and the relative ratio of calcium to phosphorus (Ca/P) was determined. The relative weight of carbon was used as background control for the calculation of relative ratio of calcium to carbon (Ca/C) and relative ratio of phosphorous to carbon (P/C).

### Microindentation analysis

Microindentation was used to test the bone local changes in elastic modulus and hardness with the modified protocols previously described in Kaya *et al*. (30). Microindentation enables the measurement of the region consisting of numerous canaliculi and at least one osteocyte nearby. Vickers hardness and elastic modulus of the bone sections were measured using a microhardness tester (DUH-211S; Shimadzu, Tokyo, Japan). The region of interest was set at the central part of trabecular bone section at least 20 µm away from the boundary. A total of 10 impressions were performed in each bone section. With modified protocol of previous reports (31), the test was conducted with a load of 100 mN for 20 s, loading speed of 6.6 mN/s, minimum force of 0.2 mN, and Poisson’s ratio of 0.200.

### Measurement of circulating bone markers

Blood was collected 1 d before the surgery. For nonsurgical subjects, blood taking was done no more than 1 mo before or after clinical assessments. Immediately after collection, clotted blood samples were processed for serum isolation by centrifugation at 3200 *g* for 10 min. Serum samples were measured in aliquotes and stored at −80°C until analysis. Serum concentrations of osteocalcin, osteopontin, dickkopf-1, and sclerostin were measured by Luminex xMAP Multiplexing technology (MilliporeSigma), whereas serum concentrations of C-terminal telopeptides (CTXs) and type I procollagen amino-terminal propeptide were measured by electrochemiluminescence immunoassay (Roche, Basel, Switzerland).

### Statistical analysis

All results were presented as means ± sd. A 2-sample Student’s *t* test was performed to compare the above parameters between 2 groups; otherwise, if the data were not normally distributed, an equivalent nonparametric Mann-Whitney *U* test was done. For the serological study, because of the significant changes in BMD and serum marker levels during puberty (32), adjustment for the *z* score of BMD at femoral head was performed in correlation analysis. BMI and body weight were not controlled because age was likely to have marked collinearity at the adolescent age group under study, which might result in inflation of type II error (33). Log_10_ transformation was conducted for data deviated from normality when necessary. ANCOVA analysis was used to compare the differences of the above parameters (dependent variables) between AIS and control (fixed factors) with controlling the age (covariate). Partial correlation with adjustment for age was applied on the correlation analysis of serum levels of bone markers with the bone phenotypes being statistically significant in ANCOVA analysis. The statistical analyses were conducted using IBM SPSS software, v.20.0 (IBM SPSS, Chicago, IL, USA). The significance level was set at *P* < 0.05 (2-tailed).

## RESULTS

### Subjects characterization

In the *ex vivo* bone tissues study, 20 patients with AIS (17 females and 3 males) and 13 control subjects without AIS (10 females and 3 males) requiring respective orthopedic surgery were recruited at our Joint Scoliosis Research Center. The mean ages of patients with AIS and control subjects were 14.3 ± 2.2 and 16.2 ± 4.8 yr old, respectively. The mean Cobb angle of patients with AIS was 55.6 ± 10.6°. It is noteworthy that patients with AIS had a slightly lower body weight and BMI despite not reaching statistical significance (*P* > 0.05), which is in line with previous reports (**Table 1**). Bone tissues from these 20 patients with AIS and 13 control subjects were used in EDX and microindentation. Among these original groups, bone biopsies from 11 patients with AIS and 11 control subjects with satisfied staining and imaging quality were used in acid-etched SEM and FITC-Imaris analysis. For this subgroup, the mean ages of patients with AIS and control subjects were 15.7 ± 1.7 and 18.0 ± 5.06 yr old, respectively. The mean Cobb angle of patients with AIS was 56.5 ± 11.4° (**Table 2**). The clinical parameters of this subgroup are comparable to those of the original group.

**TABLE 1:** Anthropometric, pubertal, and radiologic assessment in controls and AIS measured with EDX and microindentation tester

Variable	Control	AIS
Sample size (*n*)	13	20
Age (yr)*^a^*	16.5 ± 4.79	14.3 ± 2.20
Major Cobb angle (deg)*^a^*	–	55.6 ± 10.61
Arm span (cm)*^a^*	160.0 ± 13.73	158.6 ± 10.65
Body weight (kg)*^a^*	52.2 ± 17.16	46.5 ± 8.68
BMI by arm span (kg/cm^2^)*^a^*^,^*^b^*	20.1 ± 5.09	18.4 ± 2.40
Tanner stage*^c^*	3.2 ± 1.92	2.8 ± 1.54

Data are expressed as means ± sd. *^a^*Independent Student’s *t* test was used in comparison. *^b^*BMI by arm span (BMI = body weight/armspan^2^). *^c^*Mann-Whitney test was used in comparison.

**TABLE 2:** Anthropometric, pubertal, and radiologic assessment in controls and AIS measured with acid-etched SEM and FITC-Imaris technique

Variable	Control	AIS
Sample size (*n*)	11	11
Age (yr)*^a^*	18.0 ± 5.06	15.7 ± 1.74
Major Cobb angle (deg)*^a^*	–	56.5 ± 11.41
Arm span (cm)*^a^*	164.7 ± 11.96	157.6 ± 7.25
Body weight (kg)*^a^*	54.3 ± 16.33	48.3 ± 6.21
BMI by arm span (kg/cm^2^)*^a^*^,^*^b^*	19.6 ± 3.39	19.4 ± 2.05
Tanner stage*^c^*	3.7 ± 2.05	4.18 ± 0.87

Data are expressed as means ± sd.

a Independent Student’s *t* test was used in comparison. *^b^*BMI by arm span (BMI = body weight/armspan^2^). *^c^*Mann-Whitney test was used in comparison.

In the serological study, 99 girls with AIS and with a scoliosis severity from mild to severe surgical case and 31 healthy control subjects (14.3 ± 1.1 yr old) were recruited from the same center. A cutoff of Cobb angle at 45° was employed to define the surgical group (severe; Cobb angle ≥45°) or nonsurgical group (mild; Cobb angle <45°). Based on this, 27 out of 99 girls with AIS were defined as being in the severe group (15.5 ± 2.4 yr old, 65.8 ± 14.4° major Cobb angle), whereas the remaining 72 girls with AIS were defined as being in the mild group (14.8 ± 1.5 yr old, 26.6 ± 9.1° major Cobb angle). Patients with mild and severe AIS had similar age, body weight, BMI, maturity, and bone mass. In line with previous reports, patients with AIS exhibited a lower BMI compared with healthy subjects (34).

### Qualitative analysis of LCN

**Figure 1:**
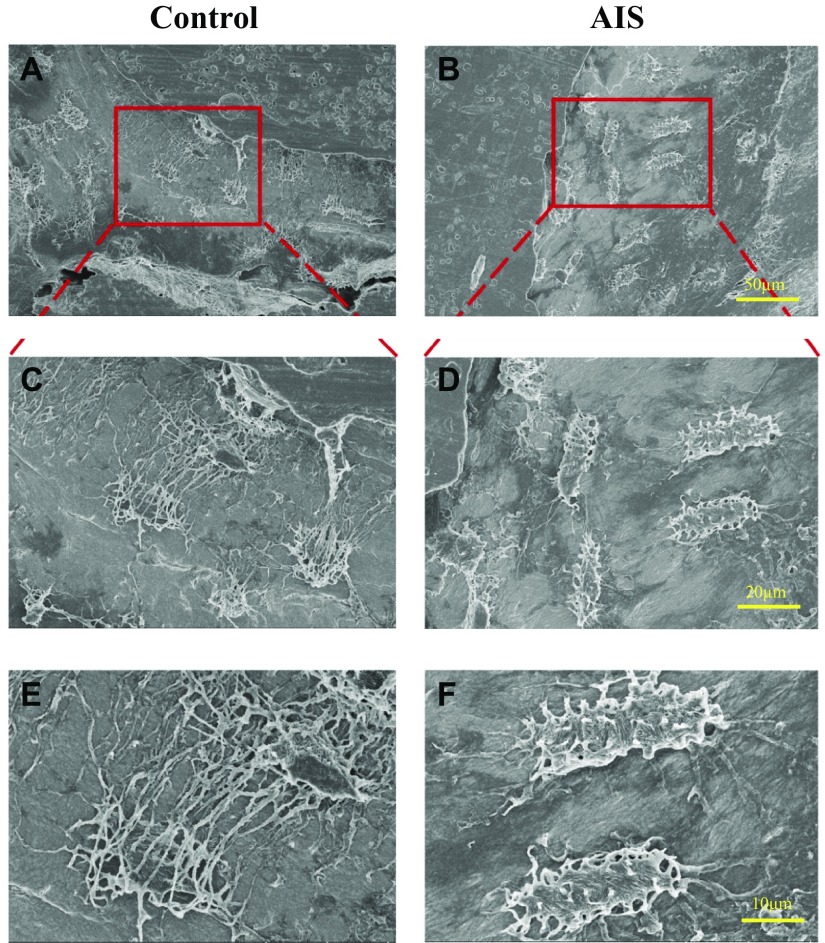
Defective structure and organization of osteocyte lacuno-canalicular walls in AIS iliac trabecular bone tissues. Osteocyte LCN (OLCN) revealed by acid-etched, resin-casted SEM imaging. Representative acid-etched SEM images showing the structure of osteocyte LCN in the iliac bone tissues from an 11.4-yr-old female (control) and a 12-yr-old girl with AIS and a similar *z* score at femoral neck BMD. *A*, *B*) Low-magnification (×400) SEM images. *C*, *D*) Medium-magnification (×1000) SEM images. *E*, *F*) High-magnification (×2000) SEM images. Note the differences between non-AIS control (left) and AIS (right) in lacunar shape, and the length and number of canaliculi.

Low-magnification (×400) SEM images show that osteocytes in the control distributed in a more organized manner. In contrast to the controls, the osteocytes in AIS bone tissues appear to be less organized in terms of distribution (**Fig. 1*A, B***). Medium-magnification (×1000) SEM images show uniform spindle-shaped osteocytes in the controls, whereas osteocytes in AIS displayed various shapes from spindle to roundish (Fig. 1*C, D*). High magnification at ×2000 shows that osteocytes in the controls were highly connected with neighboring cells *via* dense and long canaliculi protruding perpendicular from the major axis of the lacunae. In contrast, AIS osteocyte lacunae had less connectivity with fewer, shorter, and cluttered canaliculi (Fig. 1*E, F*).

### Quantitative analysis of LCN

**Figure 2:**
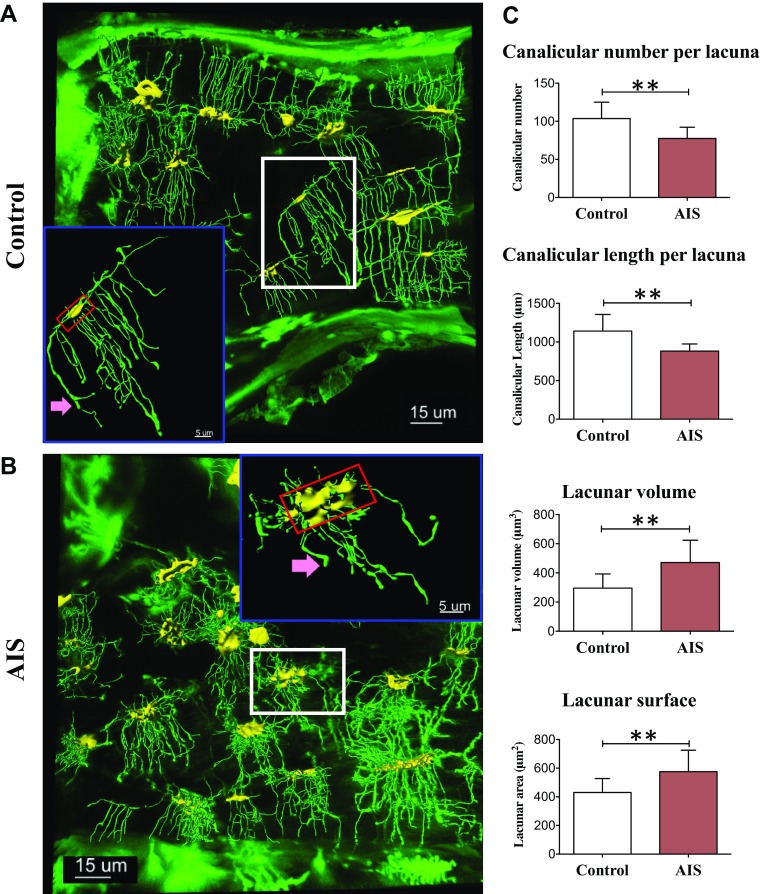
Illustration of the components of the osteocyte LCN visible in the confocal microscope superimposed with 3D LCN images constructed by Imaris software. The lacunae are highlighted as yellow masses, and the canaliculi are displayed as green lines in the Imaris 3D images. Representative Imaris-aided visualization of osteocyte cell body (yellow) and dendrite process (green) and volume rendering of FITC-stained undecalcified bone tissues with *z*-stack confocal images collected at 0.2-μm intervals. *A*, *B*) High magnification of boxed areas depicted individual LCN in the iliac bone tissues from control (*A*) and patients with AIS (*B*) (insert). *C*) Imaris-aided quantitative analysis of canalicular number and length per lacunae, lacunar volume, and area are shown. Structural parameters of osteocyte LCN, including canalicular number per lacuna, total canalicular length per lacuna, lacunar surface, and lacunar volume in iliac bone tissues from 11 controls and 11 patients with AIS were calculated by Imaris. Lacunar volume (units: µm^3^) and canalicular length (µm). Data are expressed as means ± sd. **P < 0.01.

The descriptive differences between AIS and control LCN were recapitulated by FITC-Imaris imaging technique. As shown by confocal images (**Fig. 2*A, B***), FITC penetrated the nonmineralized regions in the bone tissues, including the entire LCN, bone surface, and osteoid. Imaris was adopted to construct the 3D images of the LCN for quantitative analysis as we previously described in Ren *et al.* (29). The 3D-constructed LCN was depicted in the corresponding stacked confocal images of the bone sections. Lacunae and canaliculi were shown in yellow and green, respectively. Quantitative FITC-Imaris results were consistent with those from acid-etched SEM images. Quantitative analysis of 11 patients with AIS and 11 control subjects showed a 25% lower canalicular number (*P* = 0.003), 23% shorter canaliculi length (*P* = 0.003), 34% larger lacunar surface (*P* = 0.013), and 59% larger lacunar volume (*P* = 0.004) in patients with AIS when compared with control subjects (Fig. 2*C*).

### Bone mineral components analysis in perilacunar-canalicular region

**Figure 3:**
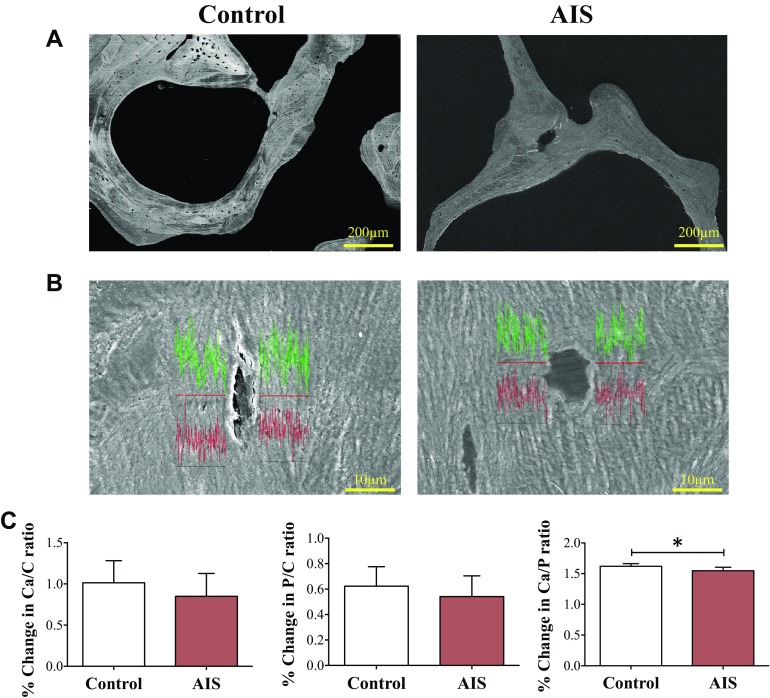
*A*) Lacunae were visualized with BSEM. *B*) Representative SEM images of the osteocyte (in yellow dashed circles) and periosteocytic regions with measurement of the elemental content of calcium (Ca) and phosphorous (P) using EDX. *B*) Calcium (Ca) and P content are depicted by green and red lines, respectively. *C*) The wt% of Ca, P, and carbon (C) were calculated by the built-in software and used for the calculation of percentage Ca/P, (Ca+P)/C, Ca/C, and P/C. Data are expressed as means ± sd. *P < 0.05.

Given that the lacunar shape has implications for the sensitivity of osteocyte to mechanosensation (35), which could be the result of osteocytic perilacunar/canalicular remodeling (36), we examined the bone mineral composition by SEM-EDX. The lacunae were visualized by BSEM (**Fig. 3*A***). Figure 3*B* illustrated the EDX element spectrum (calcium intensity in green; phosphorous intensity in red) of perilacunar-canalicular region under high-magnification SEM images (×2000) in the control and AIS bone sections. Compared with the control, the AIS perilacunar-canalicular region exhibited a significantly lower Ca/P ratio by 4.4%, whereas there were no statistical differences in calcium level (Ca/C ratio) and phosphorous level (P/C ratio) between AIS and the control (Fig. 3*C*).

### Mechanical properties analysis in perilacunar-canalicular region

**Figure 4:**
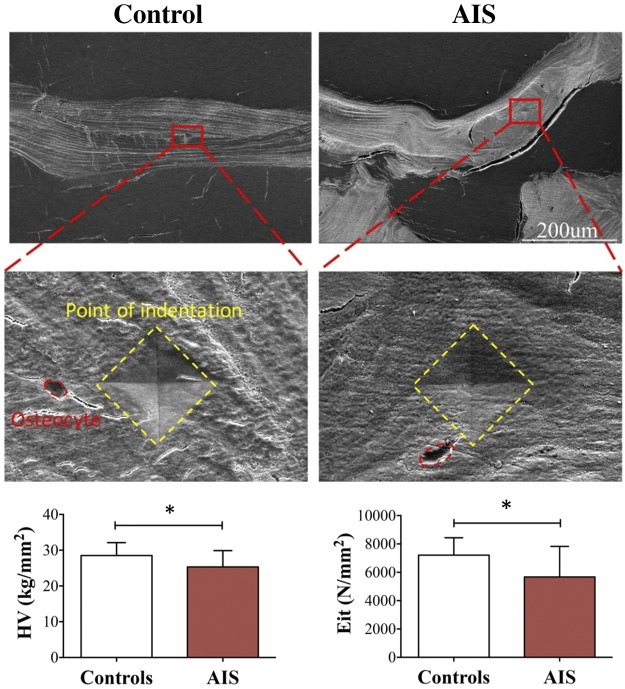
*A*) Optical micrograph of Vickers microindentation sites on MMA-embedded bone biopsy sections at the peri-osteocytic regions (osteocytes in red dashed circles and indentation sites in yellow dashed squares). Scale bars: 200 μm (top); 20 μm (bottom). *B*) Force-displacement curve of microindentation tests of control (left) and AIS (right). *C*) Comparison of Vickers hardness (HV, kg/mm^2^) and elastic modulus (Eit; N/mm^2^) between control and AIS. Data are expressed as means ± sd. *P < 0.05.

The microindentation test showed that the values of Vickers hardness and elastic modulus in the control bone tissues were 28.5 kg/mm^2^ and 7214 N/mm^2^, respectively, which were higher than that in the AIS (25.3 kg/mm^2^ and 5673 N/mm^2^, respectively). These differences were statistically significant with a mean of 11% lower hardness and 21% lower stiffness in AIS (**Fig. 4**).

### Serum bone marker measurement and correlation analysis

Based on our recent finding (20), selective serum bone markers relating to osteocyte function and bone turnover were used to investigate whether curve severity is associating with osteocyte function. The demographic, anthropometric, bone densitometric, and serum bone marker data of the 99 patients with AIS and 31 healthy control subjects are summarized in **Table 3**. The BMI corrected by arm span and aBMD at bilateral femoral necks are in agreement with previous studies (9). AIS girls with severe curve deformity (Cobb angle ≥45°) had significantly lower levels of osteocalcin and sclerostin by 16 and 12%, respectively, when compared with those in the mild group. However, a statistical difference was not found in other measured bone markers. The control group had a higher serum level of CTXs compared with mild or severe AIS groups by 25 and 49%, respectively. However, the serum osteocalcin level is statistically higher in the mild AIS group when compared with the control group by 17%. Of note, the serum sclerostin level was similar between the control and mild AIS, but statistically lower in severe AIS by 12%. **Table 4** shows Pearson correlation analysis between major Cobb angle and the serum bone markers. In the whole AIS group, the major Cobb angle correlated negatively with serum osteocalcin (*r* = −0.290; *P* = 0.003) and sclerostin (*r* = −0.197; *P* = 0.050). Subgroup analysis showed negative correlation between serum osteocalcin and Cobb angle in the severe group (*r* = −0.470; *P* = 0.015), but positive correlation in the mild group (*r* = 0.300; *P* = 0.009). Such an inverted V-shaped correlation pattern suggests the likelihood of transitional changes of osteocalcin secretion during the progression phase in AIS.

**TABLE 3:** Demographic, anthropometric, bone densitometric, and serum level of serum bone markers of control subject and patients with AIS with subgroup analysis

Variable	Severe AIS	Mild AIS	Control
Sample size (*n*)	27	72	31
Basic characteristics			
Age (yr)	15.5 ± 2.4	14.8 ± 1.5	14.3 ± 1.1*
Major Cobb angle (deg)	65.8 ± 14.1	26.6 ± 9.1	-
Anthropometric data			
Arm span (cm)	159.1 ± 6.0	156.7 ± 7.5	153.9 ± 6.8*
Body weight (kg)	46.4 ± 6.5	44.6 ± 6.8	48.3 ± 9.1
BMI by arm span (kg/cm^2^)	18.3 ± 2.1	18.1 ± 2.1**	20.2 ± 3.0*
Maturity			
Age of menarche (yr)	12.6 ± 1.4	12.5 ± 1.1	12.2 ± 1.3
Tanner stage	3.6 ± 1.0	3.6 ± 0.7	3.4 ± 1.0
Areal bone mineral density (g/cm^2^)			
Left femoral neck	0.762 ± 0.112	0.755 ± 0.071	0.80 ± 0.140
Right femoral neck	0.746 ± 0.108	0.761 ± 0.082	0.815 ± 0.132
*Z* score of BMD			
Left femoral neck	−0.295 ± 1.220	−0.497 ± 0.606	−0.060 ± 1.191
Right femoral neck	−0.518 ± 1.234	−0.493 ± 0.734**	0.073 ± 1.166
Serum bone marker			
Dickkopf-1 (pg/ml)*^a^*	1588 ± 349	1717 ± 375**	1540 ± 349
Osteocalcin (pg/ml)*^a^*	15,997 ± 12,709***	19,004 ± 7018**	15,783 ± 10,300
Osteopontin (pg/ml)*^a^*	8729 ± 7081	9136 ± 4375	10,036 ± 5270
Sclerostin (pg/ml)*^a^*	1163 ± 370***	1325 ± 393	1315 ± 505
CTX (pg/ml)*^a^*	554.9 ± 375.5	659.9 ± 445**	828.3 ± 428.2*
P1NP (μg/L)*^a^*	250.4 ± 208.7	258.8 ± 220.4	294.9 ± 246.0

P1NP, type I procollagen amino-terminal propeptide. *P < 0.05 (Student’s *t* test when comparing the parameter between severe AIS and control); **P < 0.05 (Student’s *t* test when comparing the parameter between mild AIS and control); ***P < 0.05 (Student’s *t* test when comparing the parameter between severe and mild AIS). *^a^*Log transformation was performed before Student’s *t* test.

**TABLE 4:** Pearson correlation between Cobb angle and serum bone markers in patients with AIS

	All AIS (*n* = 99)		Severe AIS (*n* = 27)		Mild AIS (*n* = 72)	
Variable	*R*	*P*	*R*	*P*	*R*	*P*
Dickkopf-1	−0.128	0.198	−0.225	0.259	0.108	0.356
Osteocalcin	−0.290	0.003*	−0.470	0.015*	0.300	0.009*
Osteopontin	−0.147	0.145	−0.382	0.054	−0.002	0.986
Sclerostin	−0.197	0.050	−0.275	0.175	0.084	0.479
CTX (pg/ml)	−0.137	0.170	−0.215	0.281	−0.104	0.376
P1NP (μg/L)	−0.036	0.722	−0.298	0.131	0.033	0.778

Log transformation on serum bone markers was performed before correlation analysis. P1NP, type I procollagen amino-terminal propeptide. *P < 0.05.

## DISCUSSION

LCN structure and organization might change with anatomic location and bone type (trabecular or cortical) (37–39). In this study, we collected trabecular iliac bone tissues from the nondominant side instead of the deformed spine where any LCN changes could be secondary to the asymmetric mechanical loading. Given that it is technically difficult to investigate a huge number of osteocytes, multiple methods were adopted to study the changes of osteocyte LCN properties at different levels. The negative correlation between curve severity and serum osteocalcin and sclerostin suggests a decreasing trend of osteocyte activity in AIS as the curve progresses. Collectively, our findings provide the first evidence of abnormal osteocyte LCN in patients with AIS and with severe curve deformity, which could be attributed to the aberrant overexpression of miRNA-145 (20).

Decreased connectivity with fewer, shorter, and cluttered canaliculi was found in AIS in contrast to the highly interconnected pattern with abundant long canaliculi radiating out in perpendicular to the long axis of the osteocytes in the control. Knothe Tate *et al.* (40) reported variations in the connectivity of the LCN in the cortical femoral neck of healthy and diseased human bone. Unlike the high connectivity of osteocytes with the dendritic processes oriented in the direction of the blood supply in normal bone, decreased connectivity of osteocytes, loss of orientation, and slack processes with higher tortuosity were found in osteoporotic bone. In osteomalacic bone, tortuous cell processes with disorganized networks could be found amid the retained connectivity. These findings indicated that the structure and organization of the LCN could reflect the underlying pathologic state of the bone tissue. Ca/P ratio was widely used as an indicator of the status of bone mineralization (41–43). Kourkoumelis *et al.* (44) have suggested that bone quality is critical for bone strength and is strongly related to the Ca/P ratio. In the present study, bone mineral component analysis showed that the Ca/P ratio was 4.4% lower in the perilacunar-canalicular region of the trabecular bone from AIS in comparison with the control (1.61 *vs.* 1.54, *P* = 0.005). Carpentier *et al.* (46) and Akesson *et al.* (44) have found that the Ca/P ratio with SEM-EDX measurements was around 1.65–1.9 in elderly subjects. However, there were no such reports for adolescents and young adults. Ca/P was reported to increase with age (46, 47); therefore, the calculated Ca/P ratios (around 1.61–1.54) in the present study were plausible. Although the microindentation method is not able to confine the measurement of tissue mechanical property at the perilacunar-canalicular region solely, our findings collectively provide evidence of abnormal osteocyte LCN in AIS.

Serum bone markers could reflect the overall bone formation and resorption processes (48). Only few studies have reported the correlation between serum bone markers and aBMD from DXA scan in AIS. Higher serum level of bone alkaline phosphatase, osteocalcin, and soluble receptor activator of nuclear factor-κB ligand and tartrate-resistant acid phosphatase serum band 5b were reported in AIS when compared with healthy control (49–52). Our data with larger sample size and controlled age showed significantly lower serum osteocalcin in the severe AIS group when compared with the mild group. Osteocalcin is a component of bone extracellular matrix produced by osteoblasts. Since the report by Ducy *et al.* (53), the determinant role of osteocalcin on bone formation has been widely acknowledged. Carboxylation of osteocalcin results in the binding to hydroxyapatite in bone (54). The serum level of intact carboxylated osteocalcin originated from new bone synthesis is a widely accepted marker for bone formation (55). Other studies suggest that the osteocalcin might have more impact on mineral maturation, thus affecting mechanical strength of bone tissue (56, 57). Being the most abundant osteoblast-specific noncollagenous protein, osteocalcin has been initially studied as a bone formation marker. However, it should be noticed that the coupling action of bone formation and bone resorption has somehow blurred the distinction of bone formation and bone resorption markers. As osteocalcin is incorporated into bone matrix, it has been suggested that fragments of carboxylated osteocalcin could be released into circulation after osteoclast-mediated bone resorption (58). The mAb used in the present study recognized intact carboxylated osteocalcin and may react with osteocalcin fragments corresponding to aa 1–19, 7–19, and 15–31 of the native molecules. A more precise analytical measurement is warranted to verify the findings of the present study. Nevertheless, the higher CTX level in the control group indicates reduced bone resorption in both mild and severe AIS groups; therefore, the higher serum osteocalcin level in mild AIS might be more likely to be attributed to increased osteoblast activity, and the drop of serum osteocalcin and sclerostin levels in severe AIS suggests the likelihood of reduced osteoblast and osteocyte activity. On the other hand, the opposite correlation patterns of serum osteocalcin at mild and severe curvature conditions further support the speculation of transitional changes in osteoblast and osteocyte activity during curve progression, leading to the abnormal osteocyte LCN in the surgical cases. Pubertal girls had negative correlation between serum osteocalcin and total BMD and spine BMD in pubertal girls (34, 59). However, we did not observe a significant correlation between serum osteocalcin and any measured bone quality parameters in severe, mild, or whole AIS (data not shown).

AIS occurs during the pubertal growth spurt. It is believed that the higher peak height velocity (the period of maximum growth rate) predisposes to a higher chance of disproportionate skeletal growth and asymmetric morphology of skeletal features (2). Despite the unclear pathomechanism on how the abnormal bone qualities could contribute to the initiation or progression, or both, of AIS, the observed close clinical association and prognostic value of low bone mass have been supported by our initial pilot studies on the effect of improving bone quality in reducing curve progression (ClinicalTrials.gov: NCT01103115). A recent proof-of-concept study demonstrated the beneficial effect of minodronate (a third-generation bisphosphonate) on reducing curve progression in a scoliosis mouse model, suggesting a novel therapeutic option for AIS (60). However, because of the unclear long-term effect of anti-osteoporotic drugs on growing adolescents, our group have also explored the clinical effect of vibration therapy on bone mass in AIS (61). A further controlled randomized trial beyond skeletal maturity is warranted to provide further definitive evidence of the sustained effect of enhanced bone qualities on decreasing curve progression to bracing or surgical threshold.

This study had several limitations. Firstly, bone tissues from AIS with mild to moderate spinal deformity were not ethically possible; therefore, the correlation between osteocyte LCN abnormalities and curve severity could not be determined. The selected serum bone marker analysis can only partly reflect the biologic roles of osteocytes in AIS. In summary, this study revealed for the first time structural and morphologic defects in AIS osteocyte LCN at tissue level. This finding is in line with previous reported studies of serum, primary osteoblast culture, and bone histomorphometry. In addition, the negative correlation between serum osteocalcin and curve severity supports the hypothesis and observation that abnormal bone metabolism, associated with impaired osteoblasts and osteocyte activity, could contribute to the etiopathogenesis and progression of AIS. The mechanism underlying abnormal osteocyte-related function warrants further in depth studies.

## Abbreviations

3D: 3-dimensional
aBMD: areal bone mineral density
AIS: adolescent idiopathic scoliosis
BMI: body mass index
BSEM: backscatter SEM
CTX: C-terminal telopeptide
DXA: dual-energy X-ray absorptiometry
EDX: energy-dispersive X-ray spectrometry
LCN: lacuno-canalicular network
SEM: scanning electron microscopy
